# Genetic polymorphisms influencing meat productivity and quality in Kalmyk cattle of Russia: A systematic review of growth hormone, thyroglobulin, leptin, and calpain-1 genes

**DOI:** 10.14202/vetworld.2026.523-538

**Published:** 2026-02-10

**Authors:** Nadezhda Chimidova, Altana Ubushieva, Victoria Ubushieva, Zanda Bochkaeva

**Affiliations:** Regional Center of Science and Production, Kalmyk State University named after B.B. Gorodovikov, 11, Pushkin str., 358000, Elista, Republic of Kalmykia, Russian Federation

**Keywords:** beef cattle genetics, *CAPN1* polymorphism, *GH* gene, Kalmyk cattle, *LEP* gene, meat quality traits, molecular markers, *TG* gene

## Abstract

**Background and Aim:**

Kalmyk cattle represent a resilient indigenous beef breed of Russia, valued for their exceptional adaptation to harsh continental climates and growing importance in sustainable beef production. Despite their economic and ecological relevance, genetic determinants underlying meat productivity and quality in this breed remain fragmented across largely regional studies. This systematic review aimed to synthesize available evidence on polymorphisms in four major candidate genes, growth hormone (*GH*), Thyroglobulin (*TG*), Leptin (*LEP*), and Calpain 1 (*CAPN1)*, and to evaluate their distribution, associations with productive traits, and relevance for marker-assisted selection in Kalmyk cattle.

**Materials and Methods:**

The review was conducted in accordance with PRISMA 2020 guidelines. A comprehensive literature search covering January 2004 to December 2024 was performed using international (PubMed, Scopus, Google Scholar) and Russian (eLibrary.ru) databases. Eligible studies included peer-reviewed articles, dissertations, and conference proceedings reporting primary genotyping data for *GH*, *TG*, *LEP*, and *CAPN1* polymorphisms in purebred or crossbred Kalmyk cattle. Data extracted included sample size, geographical origin, genotyping methods, allele and genotype frequencies, and reported genotype–phenotype associations. Due to methodological heterogeneity, a qualitative narrative synthesis was applied.

**Results:**

The synthesis revealed pronounced inter-herd and regional heterogeneity in the frequency of favorable alleles. The *GH* c.2141C>G polymorphism showed extreme variability, with the desirable VV genotype ranging from 0% to 78.3% across herds. Substantial contrasts were also observed for *LEP* polymorphisms, where favorable genotypes varied from near absence to dominance within specific populations. For meat quality markers, the *TG* c.-422C>T and *CAPN1* c.4568G>C polymorphisms displayed generally low but highly uneven frequencies. Evidence from selected breeding programs demonstrated that targeted selection can substantially increase the prevalence of desirable alleles and improve growth and carcass traits.

**Conclusion:**

Kalmyk cattle exhibit marked genetic heterogeneity for key meat productivity and quality markers, reflecting founder effects, localized selection, and breeding history. While *GH* and *LEP* polymorphisms show strong potential for marker-assisted selection, the low baseline frequency of favorable *TG* and *CAPN1* alleles highlights the need for structured, large-scale genomic strategies. This review provides the first consolidated genetic landscape of meat-related polymorphisms in Kalmyk cattle and establishes a foundation for sustainable, climate-resilient breeding programs.

## INTRODUCTION

The Kalmyk cattle (*Bos taurus*) breed, formally recognized as a distinct breed in 1934, originated during the 16th–17th centuries and is historically linked to the migration of the Oirat tribes from Western Mongolia to the Lower Volga region via present-day Kazakhstan [1, 2]. Prolonged exposure to the harsh continental climate of this region subjected Kalmyk cattle to intense natural selection, resulting in the development of distinctive adaptive traits. These animals display exceptional resilience, tolerating extreme winter temperatures reaching −40°C with strong winds and summer heat exceeding +45°C without evident adverse health effects [3]. Kalmyk breeding stock is characterized by superior heat tolerance and stable growth performance under conditions of high solar radiation and limited feed availability, highlighting its economic efficiency in marginal environments [4].

In the context of climate change, the inherent heat and drought tolerance of Kalmyk cattle [3, 4] elevates the breed from a regional asset to a genetic resource of global relevance. Strategic utilization of this resilience is critical for the development of sustainable beef production systems in arid and warming regions, thereby contributing to long-term food security. As of January 1, 2024, the breeding stock of beef cattle in the Russian Federation comprised 308.6 thousand head, of which Kalmyk cattle represented the largest proportion (33.8%), underscoring their pivotal role in the national livestock sector [5]. According to the All-Russian Research Institute of Breeding, approximately 50% of the Kalmyk cattle population is concentrated in the Republic of Kalmykia. Additional major populations occur in the Far Eastern Federal District, mainly in the Republic of Buryatia and the Trans-Baikal Territory (29.2%), and in the North Caucasus Federal District (27.2%), including the Republics of Dagestan, Kabardino-Balkaria, North Ossetia–Alania, and the Stavropol region [6].

Kalmyk cattle possess a robust constitution and pronounced homeostatic capacity, enabling stable physiological function under diverse and often extreme environmental conditions. The breed exhibits high endurance and disease resistance, with notable tolerance to thermal and nutritional stressors, traits attributed to factors such as skin and hair coat lability and localized adipose tissue distribution [7]. High resistance to infectious and non-infectious diseases, including tuberculosis, brucellosis, leukemia, and acute respiratory and intestinal disorders, has been documented [8]. Successful acclimatization and reproductive performance in northern regions of Russia, including the Republic of Sakha (Yakutia), further demonstrate their adaptive potential [9]. Genetic determinants likely underlie this innate plasticity. A genomic region on chromosome 16 (4,116,037–4,616,037 bp) containing six immune-related genes (*IL10*, *IL19*, *IL20*, *PIGR*, *FCAMR*, *IL24*) associated with infectious disease response suggests signatures of selective breeding [10]. Nevertheless, despite the identification of polymorphisms in genes related to meat productivity, direct experimental evidence remains limited, and statistically robust studies linking specific genetic polymorphisms with environmental factors are scarce. Comparative analyses of allele frequencies across ecological gradients are also largely lacking.

Beyond adaptability, Kalmyk cattle are valued for meat quality. Analyses of the longissimus dorsi muscle indicate intramuscular fat (marbling) levels ranging from 1.6% to 5.9%, showing a strong correlation with marbling scores (r = 0.97). Although total protein content in Kalmyk bull meat is marginally lower than that of Aberdeen-Angus (by 0.55%–1.95%), the protein quality index is reportedly superior [11]. Conservation and enhancement of genetic diversity are therefore essential for sustainable livestock production. Advances in molecular genetics, particularly genomic selection, have enabled precise identification and selection of animals carrying favorable alleles, resulting in measurable improvements in meat productivity and quality traits [12]. Key candidate genes associated with growth, feed efficiency, and meat quality in beef cattle include leptin (*LEP*) [13–15], thyroglobulin (*TG*) [15, 16], growth hormone (*GH*) [17, 18], calpain 1 (*CAPN1*) [19, 20], stearoyl-CoA desaturase (*SCD*) [21], diacylglycerol O-acyltransferase 1 (*DGAT1*) [22], fatty acid-binding protein 4 (*FABP4*) [23], fatty acid synthase (*FASN*) [24], and sterol regulatory element-binding protein 1 (*SREBP1*) [25].

Despite its importance, Kalmyk cattle have been investigated at the molecular level far less extensively than widely used commercial breeds. Available evidence indicates substantial genetic diversity within the breed, supported by inter-simple sequence repeat analyses [26] and microsatellite studies reporting 7–18 alleles per locus across nine markers [27]. Whole-genome single-nucleotide polymorphism analyses further revealed the presence of unique allelic combinations characteristic of indigenous, well-adapted breeds [28]. Additional studies from Russia have reinforced evidence of strong adaptive capacity and resistance to local diseases and extreme climatic conditions [29, 30].

Notwithstanding extensive zootechnical and population genetic information, a critical gap persists in the systematic synthesis of data on key polymorphisms influencing meat productivity in Kalmyk cattle. Numerous Russian-language studies have genotyped individual herds for genes such as *GH*, *LEP*, *TG*, and *CAPN1* [31–34]; however, the results are often fragmented, sometimes contradictory, and constrained by small sample sizes and predominant reliance on low-throughput methods such as polymerase chain reaction–restriction fragment length polymorphism (PCR-RFLP). Consequently, a comprehensive and critical overview that delineates the genetic landscape of the breed, reconciles inter-study inconsistencies, and identifies consistent breed-wide patterns remains lacking.

Despite the recognized importance of Kalmyk cattle as a resilient beef breed, current knowledge on the genetic basis of meat productivity and quality remains fragmented. Existing studies on *GH*, *LEP*, *TG*, and *CAPN1* polymorphisms are largely herd-specific, regionally isolated, and frequently based on small sample sizes, limiting their generalizability. Moreover, most investigations rely on low-throughput genotyping approaches, resulting in inconsistent and sometimes contradictory estimates of allele and genotype frequencies. A critical gap therefore exists in the absence of a systematic synthesis that integrates available evidence across regions and production systems, evaluates inter-herd and inter-regional heterogeneity, and clarifies the consistency of reported genotype–phenotype associations. In addition, comparative assessments linking observed genetic variation with environmental adaptation, breeding practices, or selection history in Kalmyk cattle are scarce, constraining the effective application of marker-assisted or genomic selection strategies.

The aim of this systematic review was to comprehensively synthesize and critically evaluate published evidence on polymorphisms in *GH*, *LEP*, *TG*, and *CAPN1* in Kalmyk cattle. Specifically, this study sought to (i) summarize allele and genotype frequency distributions across herds and regions of the Russian Federation, (ii) assess reported associations between these polymorphisms and traits related to growth, feed efficiency, and meat quality, and (iii) identify consistent patterns as well as sources of heterogeneity among studies. By consolidating fragmented data into an integrated genetic landscape, this review aims to define current knowledge gaps and provide a scientific foundation for future large-scale genomic studies and evidence-based breeding programs targeting sustainable improvement of meat productivity and quality in Kalmyk cattle.

## MATERIALS AND METHODS

### Ethical approval

Ethical approval was not required because this study was based exclusively on the analysis of previously published literature and did not involve live animals, biological sampling, or experimental interventions.

### Study period and location

This review included a comprehensive search and analysis conducted from July 7 through August 8, 2025, in Elista (Russia).

### Review design and reporting standards

This study was designed as a systematic review to synthesize available evidence on genetic polymorphisms associated with meat productivity and quality in Kalmyk cattle. The review was conducted and reported in accordance with the Preferred Reporting Items for Systematic reviews and Meta-Analyses (PRISMA) 2020 guidelines for systematic reviews [35].

### Protocol development and registration

A review protocol was developed a priori to define the objectives, eligibility criteria, search strategy, and analytical framework. However, the protocol was not registered in an international prospective registry. This was due to the retrospective consolidation of regionally published and non-indexed literature, particularly Russian-language sources. All methodological steps were predefined and applied consistently throughout the review.

### Review question and analytical framework

The review question was formulated using a PECO framework. The population of interest comprised Kalmyk cattle. The exposure was genetic polymorphisms in candidate genes associated with meat productivity and quality, specifically *GH*, *TG*, *LEP*, and *CAPN1*. The comparator consisted of alternative alleles or genotypes within and across herds and regions. The outcomes included growth performance, feed efficiency, carcass traits, meat quality attributes, and related phenotypic indicators.

### Literature search strategy

A systematic literature search was conducted to identify all relevant publications addressing genetic polymorphisms in Kalmyk cattle. The search covered the period from January 2004 to December 2024, with the final search completed in December 2024. Electronic searches were performed using international databases (PubMed, Scopus, and Google Scholar) and the Russian scientific database eLibrary.ru.

Search strategies combined Boolean operators (AND, OR) with general keywords and gene-specific terms in both English and Russian. Core search terms included “Kalmyk cattle” AND (“genetic polymorphism” OR SNP OR “molecular marker”). These were further refined using gene-specific combinations, including (“GH” OR “growth hormone”) AND “Kalmyk cattle” AND “Russia”, (“LEP” OR “leptin”) AND “beef cattle” AND (polymorphism OR genotype), and (“CAPN1” OR “calpain”) AND “meat tenderness” AND cattle. Database-specific syntax was adapted as required, and searches were conducted across title, abstract, and keyword fields.

Publications written in English and Russian were eligible. Reference lists of all included articles and relevant reviews were manually screened to identify additional eligible studies and minimize publication bias.

### Language and gray literature inclusion

No language restrictions were applied. English- and Russian-language publications were screened and extracted using identical eligibility criteria and data extraction procedures. Gray literature, including dissertations and conference proceedings indexed in eLibrary.ru, was eligible when full texts and primary genotyping data were available. Google Scholar results were screened systematically by relevance.

### Eligibility criteria

Studies were eligible for inclusion if they met all of the following criteria: (i) involved purebred or crossbred first filial (F1) or second filial (F2) Kalmyk cattle, (ii) reported polymorphisms, primarily single-nucleotide polymorphisms (SNPs), in *GH*, *TG*, *LEP*, or *CAPN1*, (iii) provided full-text access to peer-reviewed articles, dissertations, or conference proceedings, and (iv) reported primary genotyping results, including allele or genotype frequencies, generated using validated molecular methods, such as PCR-restriction-fragment length polymorphism (RFLP), real-time PCR, sequencing, or standardized SNP assays.

Studies were excluded if they lacked primary genotyping data, focused exclusively on other cattle breeds without direct comparative data for Kalmyk cattle, or were available only as abstracts or summaries with insufficient information for data extraction. Non-genetic studies addressing nutrition, reproduction, or morphology alone were excluded. In cases of duplicate publications, only the most complete and most recent version was retained.

### Study selection process

**Figure 1:**
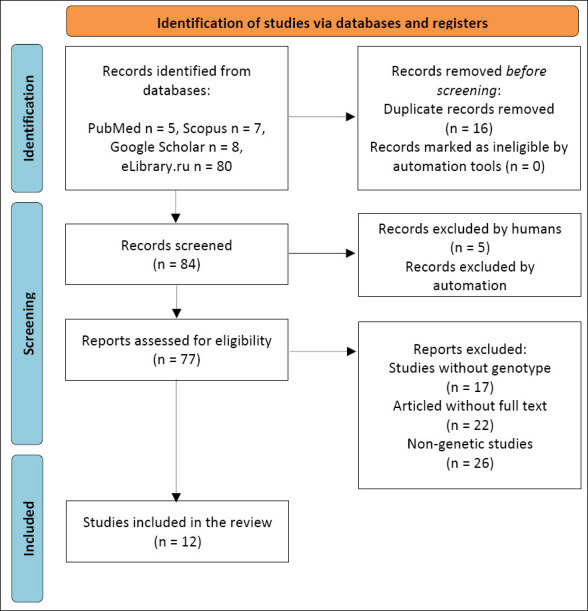
Preferred Reporting Items for Systematic Reviews and Meta-Analyses flow diagram illustrating the identification, screening, eligibility assessment, and inclusion of studies reporting genetic analyses of *growth hormone, thyroglobulin, leptin,* and *calpain-1*.

Study selection followed a multistage process consistent with PRISMA 2020 recommendations. All retrieved records were imported into a reference management system, and duplicates were removed. Titles and abstracts were screened for relevance based on eligibility criteria, followed by full-text evaluation of potentially relevant articles. The study selection process is summarized in the PRISMA flow diagram (Figure 1).

### Reviewer roles and data extraction

Study screening and data extraction were performed independently by two reviewers. Discrepancies during screening or extraction were resolved through discussion and consensus, with re-evaluation of the original publication when necessary.

Data were extracted using a predefined framework, including sample size, year of publication, geographic region and farm location, breed composition, genotyping methods, investigated polymorphisms, allele and genotype frequencies, reported genotype–phenotype associations, and applied statistical analyses (e.g., χ² test, Hardy–Weinberg equilibrium), where available. When genotype counts were reported without allele frequencies, allele frequencies were calculated.

### Quality assessment and risk of bias

Methodological quality was assessed using predefined criteria adapted for genetic association studies. Studies were evaluated for clarity in reporting animal characteristics (breed, age, sex, sample size), appropriateness and transparency of genotyping methods, completeness of allele or genotype frequency reporting, and internal consistency of results. Studies lacking critical methodological information were considered to have a higher risk of bias and were interpreted cautiously.

### Handling of population structure and heterogeneity

Substantial heterogeneity among studies was anticipated due to differences in herd structure, regional breeding practices, sample sizes, and genetic backgrounds. Population stratification, founder effects, and localized selection pressures were therefore considered during interpretation. No assumptions of population homogeneity were made across studies.

### Data synthesis and analytical approach

Due to heterogeneity in study design, genotyping platforms, investigated polymorphisms, and reported outcomes, quantitative meta-analysis was not feasible. A narrative synthesis approach was therefore applied a priori. Allele and genotype frequency distributions were qualitatively compared across studies, herds, and regions to identify trends, patterns, and pronounced differences in the prevalence of economically important alleles. Results were organized by gene and discussed thematically.

### Assessment of publication bias and certainty of evidence

Formal quantitative assessment of publication bias was not conducted because of the narrative synthesis and heterogeneous reporting formats. Potential publication bias was considered qualitatively, particularly selective reporting of significant associations in small or region-specific studies. The overall certainty of evidence was evaluated qualitatively based on sample size, consistency of findings, and methodological quality.

### Methodological limitations

Several limitations were identified across the included literature. Many studies involved small sample sizes and uneven geographic representation. A strong reliance on PCR-RFLP and other low-throughput methods was observed, with limited use of whole-genome sequencing or high-density SNP platforms. Inclusion of a substantial number of Russian-language publications was essential for regional coverage but may introduce language and publication bias. Finally, heterogeneity among studies necessitated narrative synthesis, which should be considered when interpreting the findings.

## RESULTS AND DISCUSSION

### Distribution of favorable alleles for economic traits in the population

An investigation into the allele frequencies of genes linked to economically advantageous traits in the Kalmyk cattle breed revealed substantial variations in the distribution of favorable genotypes across diverse populations and farms. Table 1 presents a summary of the genes controlling economically valuable traits in Kalmyk cattle and their frequencies [36–47].

**Table 1:** Summary of the genes controlling economically valuable traits in Kalmyk cattle.

Trait	Gene	Chr: bp (alleles)	SNP ID	Polymorphism (favorable allele)	Region	Farm	n	Breed/ Generation	Research method	Frequency of desirable genotype (%)	Ref.
Live weight gain increase	*GH*	19:48118256 C>G	rs41923484	c.2141C>G (G)	Republic of Kalmykia	LLC BR “Agrofirma Aduchi”, Tselinny district	46	Crossbred (F2)	PCR-RFLP	2.2	36
	*GH*	19:48118256 C>G	rs41923484	c.2141C>G (G)	Republic of Kalmykia	LLC BR “Agrofirma Aduchi”, Tselinny district	112	Purebred	PCR-RFLP	19.7	37
	*GH*	19:48118256 C>G	rs41923484	c.2141C>G (G)	Republic of Kalmykia	JSC BF named after A. Chapchaev, Ketchenerovsky district	60	Purebred	PCR-RFLP	78.3	38
	*GH*	19:48118256 C>G	rs41923484	c.2141C>G (G)	Republic of Kalmykia	JSC BF Kirovsky, Ketchenerovsky district	100	Purebred	PCR-RFLP	2.0	39
	*GH*	19:48118256 C>G	rs41923484	c.2141C>G (G)	Stavropol region	APC BF “Sofievsky”	16	Purebred	Real-time PCR	0	40
	*GH*	19:48118256 C>G	rs41923484	c.2141C>G (G)	Stavropol region	APC BF “Druzhba”	40	Purebred	PCR-RFLP	0	41
	*GH*	19:48118256 C>G	rs41923484	c.2141C>G (G)	Stavropol region	Breeding farms	96	Purebred	PCR-RFLP	42	42
	*GH*	19:48118256 C>G	rs41923484	c.2141C>G (G)	Stavropol region	Breeding farms	46	Purebred	PCR-RFLP	2.0	43
	*GH*	19:48118256 C>G	rs41923484	c.2141C>G (G)	Stavropol region	APC BF “Druzhba”	156	Purebred	PCR-RFLP	25	44
Meat marbling	*TG*	14:8453776 G>A	rs135751032	c.-422C>T (T)	Republic of Kalmykia	LLC BR “Agrofirma Aduchi”, Tselinny district	46	Crossbred (F2)	PCR-RFLP	6.5	36
	*TG*	14:8453776 G>A	rs135751032	c.-422C>T (T)	Republic of Kalmykia	LLC BR “Agrofirma Aduchi”, Tselinny district	112	Purebred	PCR-RFLP	13.4	37
	*TG*	14:8453776 G>A	rs135751032	c.-422C>T (T)	Stavropol region	APC BF “Druzhba”	40	Purebred	PCR-RFLP	12.5	40
	*TG*	14:8453776 G>A	rs135751032	c.-422C>T (T)	Stavropol region	Breeding farms	96	Purebred	PCR-RFLP	4.0	42
Fat accumulation, body weight, linear growth	*LEP*	4:92451008 C>T	rs29004508	c.314C>T (T)	Republic of Kalmykia	LLC PR “Agrofirma Aduchi”, Tselinny district	112	Purebred	PCR-RFLP	49.1	37
	*LEP*	4:92451008 C>T	rs29004508	c.314C>T (T)	Stavropol region	Breeding farms	96	Purebred	PCR-RFLP	1.0	42
	*LEP*	4:92451008 C>T	rs29004508	c.314C>T (T)	Stavropol region	Breeding farms	46	Purebred	PCR-RFLP	76	43
	*LEP*	4:92449032 A>T	rs29004487	c.95A>T (T)	Stavropol region	Breeding farms	46	Purebred	PCR-RFLP	83	43
	*LEP*	20:4543301 A>G	rs474894316	c.73T>C (C)	Stavropol region	APC BF “Druzhba”	156	Purebred	PCR-RFLP	29	45
Meat marbling and tenderness	*CAPN1*	29:43405875 C>G	rs17872000	c.4568G>C (C)	Republic of Kalmykia	LLC BR “Agrofirma Aduchi”, Tselinny district	46	Crossbred (F2)	PCR-RFLP	6.5	36
	*CAPN1*	29:43405875 C>G	rs17872000	c.4568G>C (C)	Republic of Kalmykia	Breeding farms	100	Purebred	Real-time PCR	20	39
	*CAPN1*	29:43405875 C>G	rs17872000	c.4568G>C (C)	Stavropol region	APC BF “Sofievsky”	16	Purebred	Real-time PCR	6	41
	*CAPN1*	29:43405875 C>G	rs17872000	c.4568G>C (C)	Stavropol region	Breeding farms	96	Purebred	Real-time PCR	3	42
	*CAPN1*	29:43405875 C>G	rs17872000	c.4568G>C (C)	Stavropol region	Breeding farms	46	Purebred	Real-time PCR	6	43
	*CAPN1*	29:43405875 C>G	rs17872000	c.4568G>C (C)	Stavropol region	APC BF “Sofievsky”	42	Purebred	Real-time PCR	5	46
	*CAPN1*	29:43405875 C>G	rs17872000	c.4568G>C (C)	Orenburg region	Breeding farms	122	Purebred	Real-time PCR	19.7	47

CAPN1 = Calpain-1 gene, GH = Growth hormone gene, LEP = Leptin gene, TG = Thyroglobulin gene, APC BF = Agricultural production cooperation breeding farm, Chr = Chromosome, F2 = Second filial, JSC BF = Joint-stock company breeding farm, LLC BR = Limited liability breeding reproducer, PCR = Polymerase chain reaction, PCR-RFLP = Polymerase chain reaction–restriction fragment length polymorphism, RFLP = Restriction fragment length polymorphism, SNP = Single-nucleotide polymorphism.

**Figure 2:**
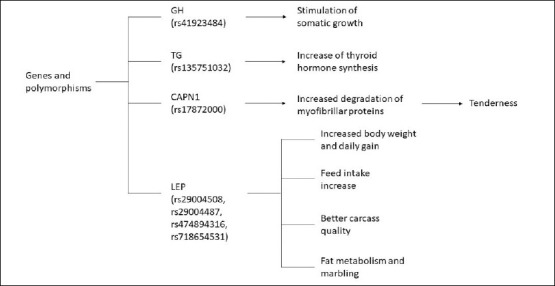
Diagram of the genes and traits discussed in the review, illustrating the relationships between growth hormone, thyroglobulin, leptin, and calpain 1 and their associated production and meat quality traits.

As illustrated in Figure 2, this review synthesizes key genes and their associated polymorphisms reported in Kalmyk cattle. For the *GH* gene (rs41923484, c.2141C>G), associated with augmented live weight gain, the favorable genotype (GG) prevalence demonstrated substantial variability, ranging from 2.0% to 78.3% among purebred herds within the Republic of Kalmykia [36–39]. A notable finding was the high frequency of the herd at Joint-Stock Company Breeding Farm (JSC BF), which is named after A. Chapchaev, which exhibited a frequency of 78.3% [38]. In contrast, the herd at JSC BF Kirovsky demonstrated a significantly lower frequency of 2.0% [39]. This pattern of high inter-herd variability was also observed in the Stavropol region, where frequencies ranged from 0% to 42.0% [40–44].

With regard to the influence of genes on meat quality traits, the distribution of favorable alleles exhibited significant heterogeneity. For the rs135751032, c.422C>T in the *TG* gene, associated with meat marbling, the frequency of the favorable TT genotype ranged from 4.0% [42] to 13.4% [37] among purebred Kalmyk cattle [36, 37, 40, 42].

The analysis of the *LEP* gene polymorphisms, which are associated with fat metabolism and growth, revealed particularly stark contrasts. For the SNP rs29004508, c.314C>T, the frequency of the desired TT genotype was 49.1% in one Kalmyk herd [37] but dropped to 1.0% in a Stavropol breeding farm [42], while another Stavropol farm exhibited an exceptionally high frequency of 76.0% for the same marker [43]. This finding suggests a significant genetic profile stratification, even among key regulatory gene.

The *CAPN1* gene has been demonstrated to influence meat tenderness and marbling, with a noteworthy finding of 19.7% in a population from the Orenburg region [47]. An analysis of gene polymorphisms was conducted, revealing the rs17872000, c.4568G>C in the *CAPN1* gene, which was studied in three regions, including Orenburg. The frequency of the desirable CC genotype exhibited a range from 3.0% [42] to 20.0% [39] across the regions under study [36, 39, 41–43, 46, 47]. The Orenburg region and the Republic of Kalmykia exhibited the highest frequency of the desirable genotype at 19.7% [47] and 20%, respectively [39], in contrast to the minimum frequency of 6.5% recorded at the LLC BR “Agrofirma Aduchi” in the Republic of Kalmykia [36] and 3% in the Stavropol region [42].

**Figure 3:**
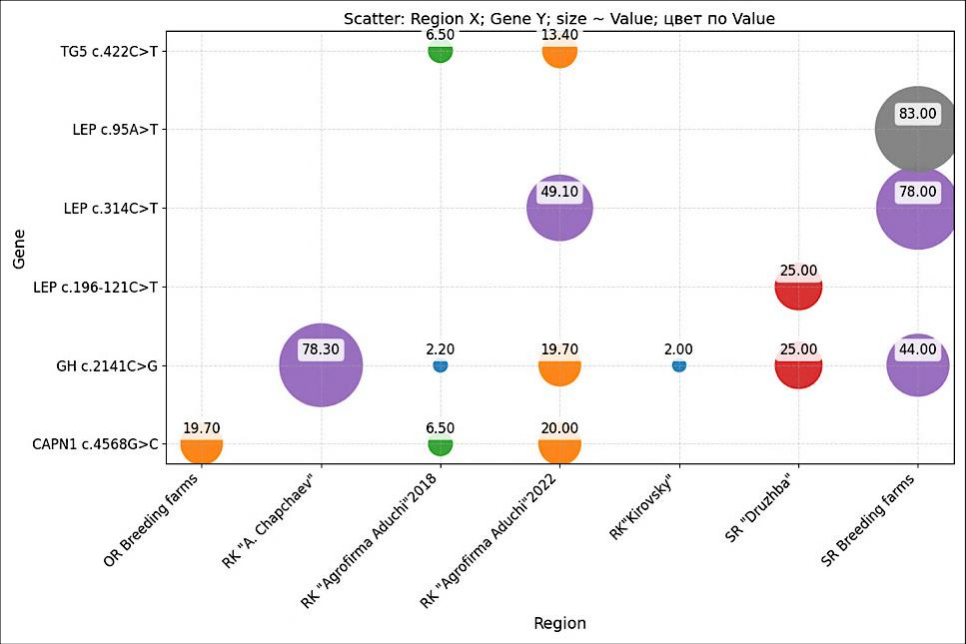
Heatmap of desirable genotype frequencies by region and gene, illustrating regional variation in polymorphisms of growth hormone, thyroglobulin, leptin, and calpain

This review provides the first integrated genetic landscape of meat quality in Kalmyk cattle, visualized as a comparative heatmap (Figure 3). The analysis qualitatively contrasts genotype frequency distributions across three major breeding regions for Kalmyk cattle: the Republic of Kalmykia, Stavropol, and Orenburg.

The previously unreported stratification of allele frequencies between herds and different regions of Russia is of significant scientific value. The analysis demonstrates that Kalmyk cattle are genetically heterogeneous, indicating the potential influence of founder effects, genetic drift, and local selection pressure.

### *GH* gene

#### Biological role and gene structure

Given that the polymorphisms in the four genes described above are considered to be the most associated with economically advantageous traits, each gene and its SNPs should be considered in more detail. *GH* is considered a principal regulatory factor for somatic growth, metabolism, and lactation in mammals. This polypeptide hormone, comprising 191 amino acids and encoded by a gene approximately 1800 base pairs in length, exerts lactogenic, lipolytic, and galactopoietic effects and significantly influences cattle reproductive performance [48–51]. The bovine *GH* gene, which is located in the 19q26 chromosomal region, comprises five exons and four introns [52, 53].

#### Major polymorphisms and functional variants

Numerous polymorphic variants have been identified within the *GH* gene. While the majority of these variants are located in non-translated intronic regions, a small number of these variants are found in exons. However, a specific C>G transversion in the fifth exon (position 2141), which results in the substitution of leucine amino acid by valine, has been identified as a primary focus of research investigating associations with productivity traits in cattle [54]. This polymorphism has been previously described in the literature as the L/V polymorphism, which indicates the amino acid transition present in the hormone. Furthermore, *GH* polymer-phisms, characterized by the substitution of CTG (allele A) to GTG (allele B) in codon 127 and ACG (alleles A and B) to ATG (allele C) in codon 172, have been demonstrated to influence carcass traits and fatty acid profile [55].

#### Single-nucleotide polymorphisms associated with economic traits

Extensive research has identified numerous other polymorphisms associated with key economic traits within *GH*. The following single-nucleotide polymorphisms have been identified: c.457C>G, linked to backfat thickness [55, 56], and c.2141C>G, a substitution caused by a polymorphism in exon 5, associated with fatty acid composition and subcutaneous fat deposition [57–60].

#### Distribution of polymorphisms within the *GH* gene

A total of 63 single-nucleotide polymorphisms (SNPs) have been identified in the bovine *GH*, with three located in the coding region and the remaining 60 in the 5′ and 3′ untranslated regions (UTRs) and introns [61].

#### Application in marker-assisted selection

The identification of polymorphisms, such as c.2141C>G, is a valuable tool for marker-assisted selection, allowing for the prediction of productivity potential and meat amino acid composition [62]. Research on Hereford bull calves has demonstrated that the *GH* c.2141C>G polymorphism significantly alters the amino acid profile of meat, although its effects are subject to breed and population specificity [63, 64].

#### GH polymorphism variability in Kalmyk cattle populations

The genetic composition of the Kalmyk cattle population with respect to the *GH* gene has been the focus of substantial research, which has revealed considerable variability. A study conducted to monitor the development of the novel “Aduchi” beef breed, presented by a crossbreeding between Kalmyk and American Aberdeen-Angus in the Republic of Kalmykia, documented an increase in the frequency of the desirable VV genotype from 2.2% (n = 46) to 19.7% (n = 112) between 2018 and 2022, indicating the efficacy of targeted selection [36, 37].

#### Comparative analyses across breeds and regions

A comprehensive comparative analysis of the *GH* c.2141C>G polymorphism across Asian steppe breeds revealed that the Kalmyk breed (n = 60) exhibited the highest frequency of the preferred VV genotype (78.3%) [38]. This result was analogous to that of the Mongolian breed (75%) and significantly higher than that of Kazakh Whiteheaded cattle of Kazakhstani (49%) and Russian (63%) selection. The minimal discrepancy between the Kalmyk and Mongolian breeds substantiates their close genetic relationship [38].

#### Low-frequency alleles and inter-herd heterogeneity

However, other studies have reported a significantly lower V allele prevalence within specific Kalmyk subpopulations. An analysis of animals from two breeding farms in Kalmykia, SPK Plodovitoye and NAO Kirovsky, found a VV genotype frequency of only 2% at both locations [39]. Despite its low frequency, VV homozygous bulls exhibited the highest live weight at 16 months, with a notable difference of 7 kg between farms, confirming a reliable association between genotype and live weight [39]. This finding of the extreme rarity of the V allele is corroborated by several other studies. Research conducted in the Stavropol Territory revealed the complete absence of the V allele, which is considered desirable, in Kalmyk cattle [40]. This finding is consistent with the results of earlier genotyping of breeding bulls, which showed a 94% prevalence of the LL genotype [41]. A subsequent investigation documented a higher frequency of the VV genotype in the Kalmyk breed, such as 42% [42]. However, a recent analysis from Stavropol breeding farms substantiated the low proportion, as only 2% of Kalmyk animals were homozygous for VV. Notably, a considerable proportion of patients were found to be heterozygous LV (43%) [43]. Another study investigating the *GH* c.2141C>G polymorphism in a population of 156 Kalmyk breed cattle in the Stavropol region established that the desirable VV genotype was present at a frequency of 25% [44].

#### Summary and transition to subsequent gene analysis

Thus, *GH* gene polymorphisms show significant variability and impact key traits in Kalmyk cattle. The role of the *TG* gene, another important marker for meat quality, is explored in the following section.

### *TG* gene

#### Biological role and functional relevance

TG is a high-molecular-weight glycoprotein synthesized in the follicular cells of the thyroid gland. It functions as a pivotal precursor and carrier molecule in the synthesis of the thyroid hormones triiodothyronine and thyroxine. These hormones play a critical role in regulating metabolism and influencing the tendency of tissues to accumulate fat [64]. The bovine *TG* gene, particularly its 5′ UTR, is one of the most extensive DNA regions in mammals. It is located in the chromosome 14 centromeric region and comprises 37 exons [65]. A well-characterized mutation in this gene, c.-422C>T, occurs in the 5′-promoter region and involves a substitution of cytosine for thymine at nucleotide position −422 [64].

#### Key polymorphisms associated with meat quality traits

The TG polymorphism X05380.1:g.-422C>T, identified in the 5′-UTR of the *TG* gene, is characterized by a C-to-T transition. This specific SNP has been used as a genetic marker to predict higher marbling scores in beef cattle, thereby enhancing meat quality. The T allele of the TG c.-422C>T polymorphism is associated with enhanced lipogenesis and reduced protein synthesis in muscle tissue. These effects are more pronounced in the homozygous state [58]. Animals with the homozygous TT genotype exhibit an augmented capacity to synthesize intramuscular fat, resulting in a substantially elevated marbling score compared with those with other genotypes [64–66].

In addition to the c.-422C>T polymorphism, further variations in this region have been identified. A thorough review of extant Chinese studies revealed four novel SNPs (c.275A>G, c.277C>G, c.280A>G, and c.281G>C) in the 5′-flanking region of the *TG* gene. The association analyses indicated that these SNPs exhibited a significant correlation with average daily gain, demonstrating a high level of significance (p < 0.01) with c.275A>G and c.277C>G and a moderate level (p < 0.05) with c.280A>G and c.281G>C [67].

#### Distribution of favorable genotypes in Kalmyk cattle

An investigation into the genetic composition of Kalmyk cattle in relation to the *TG* gene revealed a series of dynamic alterations in response to selective pressures. Research on a herd forming a new high-productivity beef type, which is a crossbreed between Kalmyk cattle and American Aberdeen-Angus in the Republic of Kalmykia, documented a notable increase in the frequency of the desirable TT genotype, from 6.5% (n = 46) in 2018 to 13.4% (n = 112) in 2022 [36, 37]. This twofold increase serves as a robust indicator of the efficacy of direct or indirect selection for traits associated with this genotype, particularly enhanced growth rate, live weight, and meat quality metrics, during the development of this novel beef type.

#### Comparative breed and regional variability

However, comparative analyses across beef breeds demonstrated considerable variation in TT frequency, underscoring population-specific differences. An investigation of genotype frequency among cattle in the Stavropol region revealed the highest prevalence of the TT genotype in Kalmyk cattle (12.5%), based on a sample of 40 animals [40]. This frequency exceeded that observed in the Kazakh Whiteheaded, Hereford, Aberdeen-Angus, and Simmental breeds at 6.3%, 0%, 3.03%, and 7.7%, respectively, with sample sizes of 16, 37, 33, and 39 animals, respectively [40].

Conversely, a separate study of herds in the same region reported a substantially lower frequency of this genotype in the Kalmyk breed, with a frequency of 4% from a sample of 96 animals [42]. This result placed it below the frequencies reported for Hereford cattle at 16% and Kazakh Whiteheaded cattle at 6% [42]. This pronounced discrepancy in reported genotype frequencies is likely attributable to factors such as geographical isolation, distinct breeding objectives and histories, and genetic drift within different subpopulations of the same breed.

Interpretation and limitations

In contrast to the *TG* gene polymorphism c.-422C>T, which is probably a key genetic marker for marbling in beef cattle, the effect of polymorphisms in other genes might be unclear.

### *LEP* gene

#### Biological role and gene structure

The *LEP* gene, which encodes the adipokine hormone leptin, plays a pivotal role in regulating energy homeostasis, feed intake, adipogenesis, and reproductive function in cattle [68]. The bovine LEP gene, which spans over 15 kilobases (kb), is located on chromosome 4q32 and comprises three exons and two introns [69, 70].

#### Functionally important polymorphisms

The polymorphism LEP c.466C>T, also known as LEP73, R4C, and R25C, is of particular interest. It is located 73 base pairs from the start of exon 2 and causes an amino acid substitution of arginine for cysteine at position 4 of the mature leptin protein [71]. The T allele of this single-nucleotide polymorphism is associated with increased leptin messenger ribonucleic acid expression. This directly influences feeding behavior, determines the highest feed efficiency, and consequently results in the formation of carcasses with high fat content. In contrast, the C allele has been linked to reduced carcass fat deposition, leading to leaner carcasses in animals carrying this genotype [72].

The LEP c.528T>C polymorphism is located in the 5′ UTR and has been shown to have a positive influence on marbling and meat yield in beef cattle. Additionally, the study describes the LEP c.73 polymorphism T>C, which is located in exon 2, has been shown to have a positive effect on adipose tissue formation and carcass quality [51, 73, 74].

Polymorphism c.95A>T, also known as Y7F, is located in the coding part of the *LEP* gene within exon 2, resulting from the nucleotide substitution A>T at position 95,689,996 bp [75]. The paucity of research on this subject is primarily attributable to the rarity of non-YY genotypes within populations [76]. Researchers in Ireland have demonstrated a correlation between the LEP Y7F polymorphism in cattle and increased energy accumulation in the form of adipose tissue deposits [74]. In contrast, Japanese studies have identified a relationship between this polymorphism and its influence on carcass weight [77].

Significant associations between novel exonic SNPs and enhanced carcass traits in Chinese Simmental cattle have been identified. These SNPs, specifically 169T>C and 299T>A in exon 2 and exon 3, respectively, have been shown to impact characteristics such as slaughter weight, marbling score, and intramuscular fat [78]. A subsequent review of polymorphisms associated with beef traits emphasized the role of LEP SNPs in meat flavor, fat thickness, and meat yield [79].

#### Evidence from Kalmyk cattle populations

The most comprehensive genotyping study in Kalmyk cattle (n = 156) identified c.73T>C as a key LEP polymorphism [45]. Allele frequencies for c.73T>C were C: 0.52 and T: 0.48. Robust association analyses were conducted: individuals heterozygous CT or homozygous CC for the c.73T>C polymorphism exhibited a 7.3% and 9.5% greater live weight, respectively, than TT homozygous counterparts [45].

The genetic architecture of the LEP locus in Kalmyk cattle demonstrates considerable population-specific variation, reflecting diverse breeding histories and selection pressures. An investigation of a novel high-productivity crossbreed beef type of Kalmyk and Aberdeen-Angus in the Republic of Kalmykia revealed a high prevalence of the TT genotype for the c.314C>T polymorphism; therefore, 49.1% out of 112 animals were TT homozygous, suggesting that this allele was selected for active or inadvertent selection during herd formation [37]. The polymorphism is frequently characterized by an amino acid change at position A80V, resulting in the following genotypes: AA, AV, and VV.

The highest frequency of reliable genotypes was observed in the Kalmyk cattle population from the Stavropol region, with a sample size of 46. In this group, 79% of the animals possessed the AA genotype at the c.314C>T locus, whereas 83% were homozygous for the YY genotype at the c.95A>T position [43]. In stark contrast, a study of 96 Kalmyk animals from the Stavropol region reported a markedly low frequency, with only 1% having a desirable LEP TT haplotype at position 140 of exon 3, which was considerably more common in contemporary Hereford (12%) and Kazakh Whiteheaded (10%) populations from the same region [42]. This discrepancy underscores the presence of significant genetic stratification within the Kalmyk breed, likely attributable to geographical isolation and divergent breeding objectives.

#### Interpretation and implications

These findings provide compelling evidence of a significant association between specific LEP alleles and growth traits in Kalmyk cattle. However, the contradictory results regarding allele frequencies across studies highlight the lack of population homogeneity and preclude a definitive consensus. Consequently, while the LEP gene represents a promising candidate for marker-assisted selection in Kalmyk cattle, further validation in larger, well-defined cohorts is essential to confirm its utility and to account for population substructure, genotype-by-environment interactions, and potential epistatic effects before its implementation in breeding programs.

### *CAPN1* gene

#### Structure and functional relevance

The *CAPN1* gene, located on chromosome 29, encodes the catalytic large subunit of the μ-calpain protease, a calcium-dependent enzyme critically involved in post-mortem proteolysis and meat tenderization [80]. The bovine *CAPN1* gene comprises 19 exons and 17 introns spanning 11,055 base pairs [81]. Several polymorphisms within this gene have been extensively characterized for their impact on meat quality traits [82].

#### Key polymorphisms associated with meat tenderness

Several SNPs within *CAPN1* are considered key molecular markers for meat tenderness. These include substitutions at codon 316 (c.4568G>C, Gly/Ala) in exon 9 [83, 84], codon 530 (c.654A>G, Ile/Val) in exon 14 [84], and intronic polymorphisms at positions 4751 and 5331 [85]. Meta-analyses have consistently identified the C allele of 316G>C, the G allele of 530A>G, and the C allele at position 4751 as favorable markers associated with reduced shear force and improved meat tenderness [58, 83]. Among these, the 316G>C polymorphism in exon 9 exerts the most pronounced effect on meat tenderness across diverse cattle breeds [86, 87]. The C allele is considered advantageous as it significantly improves meat tenderness [88].

#### Functional significance of the c.4568G>C (rs17872000) polymorphism

Within the coding region of *CAPN1*, a nonsynonymous nucleotide substitution g.43405875C>G results in the replacement of glycine with alanine at amino acid position 316 (rs17872000). The C316 allele is considered the desirable allelic variant associated with increased meat tenderness. Animals homozygous for this allele are of particular interest for selection aimed at improving meat quality. Cattle carrying the alanine variant demonstrate markedly higher meat tenderness compared with carriers of the glycine allele, with reported improvements exceeding 30%.

The biological mechanism underlying this association involves the role of μ-calpain in post-mortem degradation of myofibrillar proteins, particularly Z-disks, leading to weakening of sarcomere structures and enhanced tenderization [89–91].

#### Distribution of *CAPN1* polymorphisms in Kalmyk cattle

Investigations into the *CAPN1* genetic structure of Kalmyk cattle reveal substantial heterogeneity and an overall low prevalence of favorable alleles. In a newly developed high-productivity crossbred beef type of Kalmyk and Red Angus in the Republic of Kalmykia, the frequency of the preferred CC genotype ranged from 3% to 12%, with a strong predominance of the GG genotype (69%–77%) [36]. Similar patterns were observed in the Stavropol region, where the CC genotype frequency was only 5% among 42 Kalmyk animals [46], comparable to Hereford (5%) and Kazakh Whiteheaded (6%) cattle from the same region. Other studies reported CC genotype frequencies of 6% in small Kalmyk cohorts (n = 16 [41] and n = 46 [43]) and 3% in a larger sample of 96 animals [42].

#### Inter-herd variation and regional stratification

Pronounced subpopulation stratification has been identified within the Kalmyk breed. A comparative analysis of two breeding farms in Kalmykia, SPK Plodovitoye and NAO Kirovsky, revealed higher frequencies of the desirable c.4568G>C CC genotype at SPK Plodovitoye compared with NAO Kirovsky (22% vs. 18%). Bulls from SPK Plodovitoye carrying the favorable genotype also exhibited a higher live weight at 16 months (489.3 kg), exceeding those from NAO Kirovsky by 7.1 kg, suggesting a potential pleiotropic effect or linked selection for growth traits [39]. In the Orenburg region, a relatively higher CC genotype frequency (19.7%) was reported among 122 Kalmyk cattle, exceeding that observed in other local breeds [47], highlighting the influence of regional breeding strategies on allele distribution.

#### Implications for marker-assisted selection

In conclusion, while *CAPN1* is a well-established molecular marker for meat tenderness in global beef cattle populations, its practical application in Kalmyk cattle breeding programs is constrained by the low overall frequency of favorable alleles and marked inter-herd variability. Associations between *CAPN1* genotypes and economically important traits in Kalmyk cattle require further validation in larger, well-controlled cohorts to account for population structure and environmental interactions before reliable implementation in marker-assisted selection strategies.

## CONCLUSION

This systematic review provides an integrated synthesis of available evidence on genetic polymorphisms influencing meat productivity and quality in Kalmyk cattle. Across studies, substantial inter-herd and inter-regional variability was consistently observed for favorable genotypes of *GH*, *TG*, *LEP*, and *CAPN1*. Polymorphisms in GH (c.2141C>G) showed strong associations with live weight and growth performance, whereas TG (c.-422C>T) emerged as a key determinant of marbling and intramuscular fat deposition. Variants in LEP were repeatedly linked to growth rate and fat metabolism, although allele frequencies differed markedly among subpopulations. In contrast, favorable *CAPN1* alleles associated with meat tenderness were generally rare, despite clear phenotypic advantages where present. Collectively, these findings demonstrate that Kalmyk cattle represent a genetically heterogeneous breed with considerable untapped potential for meat quality improvement.

The pronounced variability in allele and genotype frequencies across regions indicates that uniform breeding strategies are unlikely to be effective. Instead, region-specific and herd-level selection programs incorporating *GH*, *TG*, *LEP*, and *CAPN1* markers could substantially enhance selection efficiency. The documented response to selection in crossbred and improved Kalmyk populations highlights the feasibility of integrating molecular markers into practical breeding schemes. These insights are particularly relevant for sustainable beef production systems in harsh and variable environments, where Kalmyk cattle already demonstrate strong adaptive advantages.

A major strength of this review lies in the systematic consolidation of fragmented literature, including region-specific studies that are often excluded from global syntheses. By integrating genotype distributions, association results, and regional comparisons, this review provides the first coherent genetic landscape of meat productivity traits in Kalmyk cattle. The gene-focused synthesis enables direct comparison of evidence across studies while maintaining biological and breeding relevance.

Several limitations should be considered when interpreting the findings. Many studies were characterized by small sample sizes and uneven geographic representation, which may limit generalizability. The predominance of low-throughput genotyping methods restricted haplotype-level and genome-wide inference. In addition, heterogeneity in study design, phenotypic measurements, and statistical approaches precluded quantitative meta-analysis, necessitating a narrative synthesis. Population stratification and genotype-by-environment interactions were not consistently accounted for across studies.

Future research should prioritize large-scale, well-designed studies incorporating high-throughput genotyping and whole-genome approaches to validate candidate polymorphisms and identify novel loci. Integrating genomic data with detailed phenotypic, environmental, and management information will be essential to disentangle population structure effects and improve predictive accuracy. Longitudinal studies assessing correlated responses to selection and gene–environment interactions would further support the sustainable application of marker-assisted and genomic selection in Kalmyk cattle.

In conclusion, *GH*, *TG*, *LEP*, and *CAPN1* represent biologically meaningful and economically relevant genetic markers for meat productivity and quality in Kalmyk cattle. While clear associations have been identified, their practical utility depends on population context, allele frequency, and breeding objectives. This review establishes a critical foundation for evidence-based genetic improvement and underscores the need for coordinated, genomics-enabled breeding strategies to fully realize the genetic potential of this resilient beef breed.

## DATA AVAILABILITY

All data analyzed in this review were derived from previously published studies. No new datasets were generated. Extracted data are presented within the article tables and figures.

## AUTHORS’ CONTRIBUTIONS

NC and ZB: Designated and conducted the study and drafted and edited the manuscript. AU and CU: participated in data collection and discussion. All authors have read and approved the final version of the manuscript.

## COMPETING INTERESTS

The authors declare that they have no competing interests.

## PUBLISHER’S NOTE

Veterinary World remains neutral with regard to jurisdictional claims in the published institutional affiliations.
